# Maternal infection and risk of intrapartum death: a population based observational study in South Asia

**DOI:** 10.1186/1471-2393-13-245

**Published:** 2013-12-28

**Authors:** Azusa Iwamoto, Nadine Seward, Audrey Prost, Matthew Ellis, Andrew Copas, Edward Fottrell, Kishwar Azad, Prasanta Tripathy, Anthony Costello

**Affiliations:** 1 Bureau of International Medical Cooperation, Japan, National Center for Global Health and Medicine, Medicine 1-21-1, Toyama, Shinjuku-ku, Tokyo, Japan; 2 UCL Institute for Global Health, University College London, 30 Guilford St, London WC1N 1EH, UK; 3 Centre for Child and Adolescent Health, School of Social and Community Medicine, Oakfield House, Oakfield Grove, University of Bristol, Bristol BS28 2BN, UK; 4 UCL Centre for Sexual Health and HIV Research, Institute of Epidemiology & Health Care, University College London, London, UK; 5 Perinatal Care Project, Bangladesh Diabetic Samity, Diabetic Association of Bangladesh, Dhaka, Bangladesh; 6 Ekjut, Ward, Number 17, Plot 556B, Potka, Po-Chakradharpur, District West Singhbhum, Jharkhand 833102, India

**Keywords:** Maternal infection, Double hit hypothesis, Intrapartum-related neonatal death, Prolonged rupture of membranes, Neonatal mortality, Low-income countries, Resource-poor

## Abstract

**Background:**

Approximately 1.2 million stillbirths occur in the intrapartum period, and a further 717,000 annual neonatal deaths are caused by intrapartum events, most of which occur in resource poor settings. We aim to test the ‘double-hit’ hypothesis that maternal infection in the perinatal period predisposes to neurodevelopmental sequelae from an intrapartum asphyxia insult, increasing the likelihood of an early neonatal death compared with asphyxia alone. This is an observational study of singleton newborn infants with signs of intrapartum asphyxia that uses data from three previously conducted cluster randomized controlled trials taking place in rural Bangladesh and India.

**Methods:**

From a population of 81,778 births in 54 community clusters in rural Bangladesh and India, we applied mixed effects logistic regression to data on 3890 singleton infants who had signs of intrapartum asphyxia, of whom 769 (20%) died in the early neonatal period. Poor infant condition at five minutes post-delivery was our proxy measure of intrapartum asphyxia. We had data for two markers of maternal infection: fever up to three days prior to labour, and prolonged rupture of membranes (PROM). Cause-specific verbal autopsy data were used to validate our findings using previously mentioned mixed effect logistic regression methods and the outcome of a neonatal death due to intrapartum asphyxia.

**Results:**

Signs of maternal infection as indicated by PROM, combined with intrapartum asphyxia, increased the risk of an early neonatal death relative to intrapartum asphyxia alone (adjusted odds ratio (AOR) 1.28, 95% CI 1.03 – 1.59). Results from cause-specific verbal autopsy data verified our findings where there was a significantly increased odds of a early neonatal death due to intrapartum asphyxia in newborns exposed to both PROM and intrapartum asphyxia (AOR: 1.52, 95% CI 1.15 – 2.02).

**Conclusions:**

Our data support the double-hit hypothesis for signs of maternal infection as indicated by PROM. Interventions for pregnant women with signs of infection, to prevent early neonatal deaths and disability due to asphyxia, should be investigated further in resource-poor populations where the chances of maternal infection are high.

## Background

Approximately 1.2 million of the world’s annual 3 million stillbirths occur in the intrapartum period, and a further 717,000 of 3.1 million annual neonatal deaths are caused by intrapartum events [1-4]. Until recently, these neonatal deaths were broadly classified as caused by ‘birth asphyxia’, but recent guidance recommends using the term ‘intrapartum-related neonatal death’, defined as the death of a term infant with neonatal encephalopathy, or who cannot be resuscitated [1]. The burden of morbidity associated with intrapartum-related asphyxial events is also high: according to the World Health Organisation’s global disease burden estimates, ‘birth asphyxia’ was responsible for an estimated 42 million disability adjusted life years (DALYs) in 2004, making it the eighth leading cause of disease for all age groups [5].

Several community-based studies have provided data on intrapartum-related neonatal deaths in developing countries using verbal autopsies [1,6-11]. A recent study from Matlab, Bangladesh, showed that intrapartum-related events were responsible for 53% of early neonatal deaths and approximately three percent of late neonatal deaths [7]. In South Asian communities, the intrapartum-related neonatal mortality rate is estimated at between nine and 15 per 1000 live births in the absence of intervention [8].

Previous studies have shown that clinical markers of maternal infection such as pyrexia and chorioamnionitis are significant risk factors for neonatal encephalopathy, low Apgar score and neurological outcomes such as cerebral palsy [12-15]. In Nepal, a facility-based study found that prolonged rupture of membranes (PROM), meconium stained amniotic fluid and thick meconium were important risk factors for encephalopathy among term newborn infants [16]. A prospective community-based study in rural Nepal also found maternal fever to be a significant risk factor for birth asphyxia [11]. Some studies have suggested that the combination of maternal infection and neonatal encephalopathy increases the likelihood of adverse neurological outcomes including the risk of cerebral palsy [17,18].

The ‘double hit hypothesis’ is an example of a causal pathway approach to irreversible neonatal brain injury and neonatal death, focusing on perinatal maternal infection and intrapartum events. It hypothesises that the first insult (maternal infection) renders the perinatal brain more vulnerable to the second (asphyxial) insult [17-19]. Other evidence in support of the double hit hypothesis is based on raised blood levels of pro-inflammatory mediators in term infants who have experienced respiratory arrest and brain injury following intrapartum compromise in association with maternal chorioamnionitis [20-22].

In this study we aimed to test the hypothesis that intrapartum compromise affecting infants born to mothers with clinical signs of infection immediately prior to, or during delivery, is more likely to result in an early neonatal death compared with compromised infants born to mothers without evidence of infection. This is the first population-based study to investigate the ‘double hit hypothesis’ in a low-resource setting.

## Methods

### Study populations and interventions

**Figure 1:**
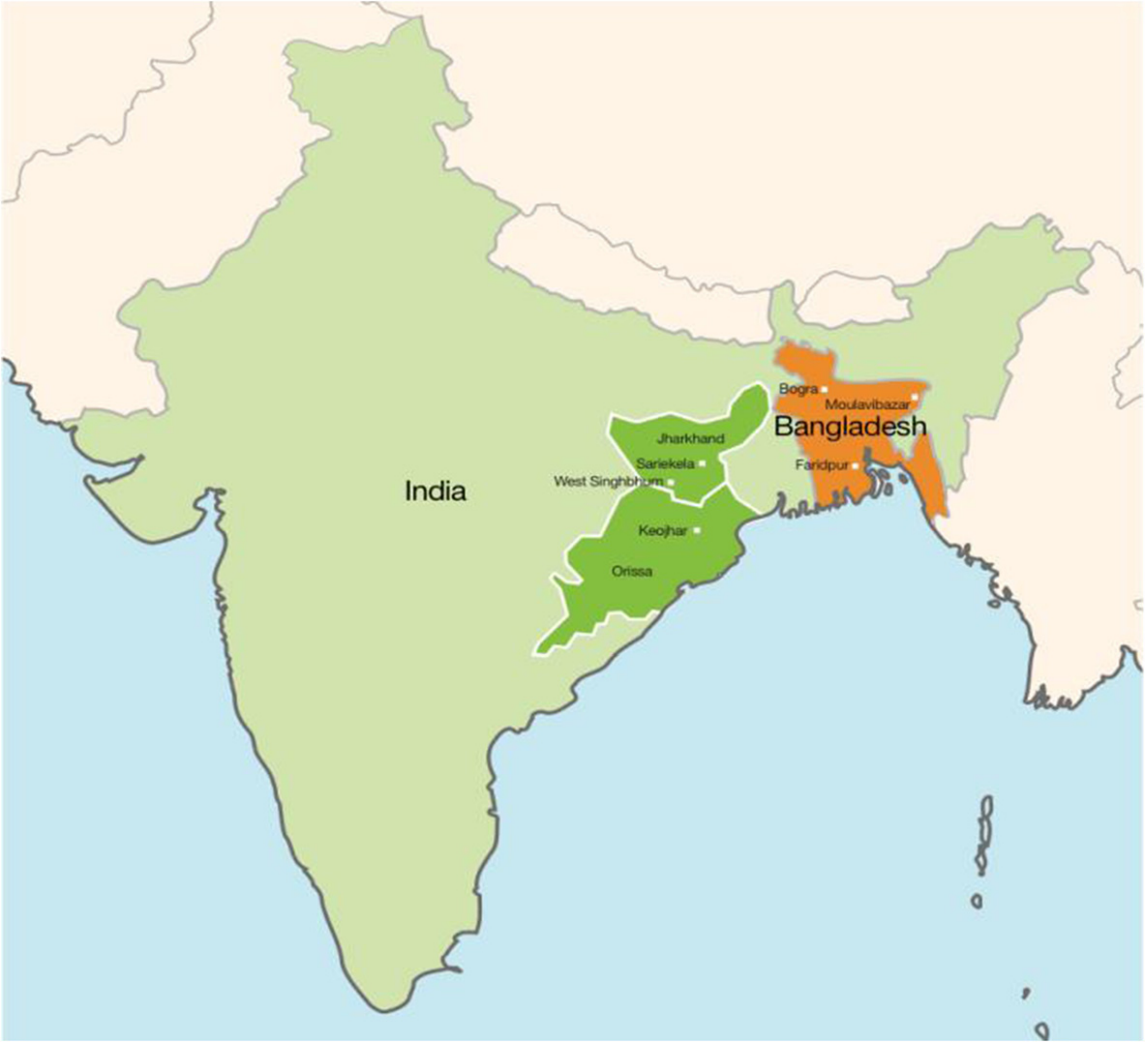
Map displaying the location of the different study sites.

We used data from the intervention and control arms of three community-based cluster-randomised controlled trials (cRCTs) carried out between 2005 and 2011 in Bangladesh (two cRCTs) and India (one cRCT) [23-26]. Figure 1 shows the study locations. Intervention clusters received a community-based participatory intervention with women’s groups aimed at improving maternal and newborn health. Table 1 describes the characteristics of these studies. As rates of maternal infection and intrapartum related asphyxia were similar in both intervention and control groups, we felt it was appropriate to include participants from both study arms. Additionally, results from a two way interaction term indicated that intervention and control groups were similar in the effect of the exposure on early neonatal death.

**Table 1:** Characteristics of the studies and populations included in the analyses

**Characteristics**	**Bangladesh**	**India**
Location	Three districts: Bogra, Maulvibazaar, and Faridpur	Three districts of Jharkhand and Orissa (eastern India): Keonjhar, West Singhbhum, and Saraikela
Study period	Feb, 2005 to June, 2011	July, 2005 to July, 2010
Study design	1st cRCT, factorial design, open cohort between February 2005 and December 2007.	cRCT, open cohort between 2005 and July 2010.
	2. 2nd cRCT, open cohort between Jan 2009 to June 2011.	
Cluster characteristics	Villages making up a union	8–10 villages with residents classified as tribal or other backwards caste.
Number of clusters analysed	18	36
Participants	Women aged between 15 and 49 y who had given birth in study period and their infants.	Women aged between 15 and 49 y who had given birth in study period and their infants.
Early neonatal mortality rate prior to intervention (per 1,000 live births)	33	40
Maternal signs of infection available for analysis	Fever and PROM	Fever

**Figure 2:**
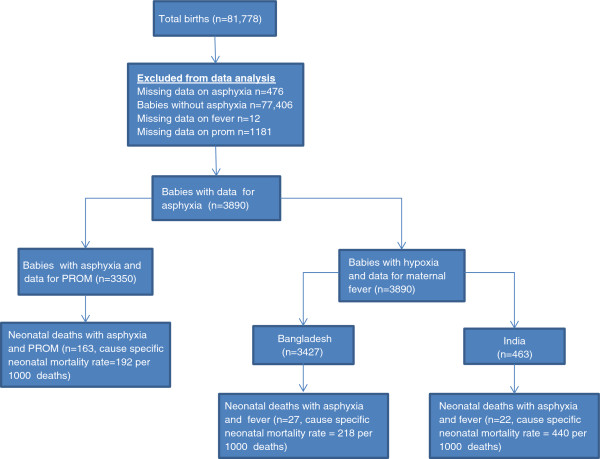
**Flowchart on inclusion criteria for study population, detailing missing data and other exclusion criteria.** Description: This is a table detailing comparisons between one of our main exposure groups of maternal fever. This table was quite long and we were not able to incorporate into the main document.

We included data from infants with signs of intrapartum asphyxia born either at home or in health facilities, and with complete data on both intrapartum asphyxia and maternal infection. Multiple births (twins and triplets) were excluded as these infants are known to be at increased risk for intrapartum-related neonatal death and probably not representative of the exposure of maternal infection. The numbers of infants included for each exposure examined, including the total sample size before and after exclusions, are shown in Figure 2.

### Surveillance systems and exposure and outcome ascertainment

This study’s main outcome was early neonatal death, defined as a death occurring in the first seven days of life [27]. Early neonatal death was chosen rather than neonatal death as most deaths due to intrapartum-related events occur in the first seven days of life [5]. The two study sites had similar surveillance systems to monitor vital events and information. Briefly, after a key informant had identified a birth, neonatal death or stillbirth, a trained field worker visited the mother or appropriate family member in the community up to six weeks after birth to administer a structured questionnaire collecting information on socio-demographic characteristics as well as events during pregnancy, delivery, and the postpartum period. In the case of a neonatal death, a detailed verbal autopsy questionnaire, separate to the surveillance questionnaire, was administered to the mother by the fieldworker. Information about the individual surveillance systems can be found elsewhere [23-26].

Data collected through the above-mentioned surveillance systems contained information on exposures of interest including a combination of the clinical manifestations of maternal infection and intrapartum asphyxia. The following signs of maternal infection were used to determine exposure status: fever up to three days prior to labour and prolonged rupture of membranes (PROM) greater than twenty four hours prior to delivery. These clinical signs are realistic markers of maternal infection [28]. Data on maternal fever were collected at both study sites using identical questions, but data on PROM were collected only in Bangladesh.

The study population included infants with signs of intrapartum asphyxia, present five minutes after delivery. In both India and Bangladesh similar questions were also administered, asking the respondent to describe the condition of the infant at five minutes after delivery as being ‘good’ when the infant was reported to be “*crying or breathing well, pink, and active*;” ‘poor’ when “*breathing poorly with blue limbs and little activity*;” and as ‘bad’ when there was reported to be “*no cry, blue body, and no movement*.” We included infants in ‘*poor*’ or ‘*bad*’ condition at five minutes as a proxy for possible intrapartum asphyxia. Neonatal condition at five minutes has been used elsewhere to report intrapartum related injuries, although this measure has not been validated using data from this particular survey questionnaire [8].

### Data collection and management

Data were collected on paper, entered by separate data entry operators and cross-checked by data managers. Databases were created in Microsoft Access or SQL Server. Quality control processes have been described previously [23,24,26].

### Statistical methods

Data were analysed and reported, including missing data and sensitivity analyses according to the STROBE recommendations [29]. We carried out univariable analyses using the combined data from Bangladesh and India, and then separately for each site, to compare outcomes for newborn infants with evidence of intrapartum asphyxia and maternal signs of infection with those among asphyxiated neonates without maternal infection. We examined potential confounders and effect modifiers using chi-squared tests or Fisher’s exact tests where appropriate, with a p-value <0.05 considered significant. Confounders were carefully considered based on *a priori* knowledge of their associations with neonatal death, intrapartum asphyxia and maternal infection. These included maternal education, parity, maternal age, number of antenatal visits, malpresentation at delivery, type of delivery (vaginal, assisted vaginal or cesarean), household assets (all assets include those households containing any one of the following items; television, fridge, electricity; some assets refer households having any one of the following; a bicycle, radio, fan or phone, and no assets refer to a household not having any of the above mentioned assets), delivery by a skilled birth attendant (i.e. doctor, nurse or trained midwife), delivery at a healthcare facility, and whether the mother lived in women’s group intervention or control areas. Maternal age, parity, and number of antenatal care visits were treated as discrete variables whereas the remaining variables were categorical.

Respondents with missing information on either maternal infection or intrapartum asphyxia were compared to those respondents with complete responses using the chi-squared test or Fisher’s exact test, where appropriate, to determine if these missing data could potentially bias our subsequent findings.

To determine whether the effect of maternal infection on early neonatal mortality was different in the intervention and control arms, an interaction term was fitted between a dummy variable representing intervention or control allocation and the different exposures for maternal infection (i.e. PROM and maternal fever). Results from the interaction term (p > 0.05) indicate that the effect of the exposure on the outcome was similar for the treatment and control arms, suggesting that pooling data from these two strata is appropriate.

We used mixed effect logistic regression models to test our hypothesis. We tested for associations between each of the clinical signs of maternal infection combined with intrapartum asphyxia with early neonatal death separately, after adjusting for all potential confounders listed earlier and, when using the combined data, adjusting also for study site.

To check the credibility of our findings, we categorised early neonatal deaths as asphyxia-related or not using verbal autopsy data. Cause-specific classification was achieved by processing the data made available from the verbal autopsy questionnaire, through InterVA version 4.02 (http://www.interva.net), a probabilistic method that estimates the probability of specific causes of death based on reported signs, symptoms and circumstances derived through verbal autopsy [30]. Early neonatal deaths were classified as asphyxia-related if InterVA identified asphyxia as one of the three most likely causes of death. Asphyxia-specific early neonatal deaths were then used as the outcome measure and associations between clinical signs of maternal infection were explored using similar adjusted mixed effect logistic regression models as was done in the main analysis.

As data was collected from 54 geographical clusters (18 in Bangladesh and 36 in India), there was potential for neonatal mortality to be correlated within clusters. Likelihood ratio statistics were used to test for such correlation in the data. Significant intra-cluster correlation was found and mixed effect models were used to account for this. The data were analysed using STATA version 12.0 (Stata Corporation, College Station, Texas, USA) [31].

### Ethical approval

Ethical approval for the cRCTs and subsequent data collection came from the Institute of Child Health and Great Ormond Street Hospital for Children (UK) and the following in-country research ethics committees: the ethics committee of the Diabetic Association of Bangladesh (Perinatal Care Project, Bangladesh Diabetes Society or BADAS) and an independent ethics committee in Jamshedpur, India (Guided by the Indian Council of Medical Research (ICMR) Guidelines of 2006 whose ambit is National). All participants gave consent to be interviewed in writing, by thumbprint or verbally.

## Results

### Study population characteristics

Before excluding newborns without signs of intrapartum asphyxia, the sample size was 81,778 (63,985 in Bangladesh and 17,793 in India). The early neonatal mortality rate for the total study population was 23 per 1000 live births, (21 in Bangladesh and 29 in India). Signs of intrapartum asphyxia were present in 4.8% live newborns five minutes after delivery (5.4% in Bangladesh, 2.6% in India). Maternal fever in the three days prior to delivery was reported for 2.3% of all deliveries, (1.7% in Bangladesh and 4.8% in India). Signs of maternal PROM were reported for 16.6% of deliveries in Bangladesh.

After exclusions, there were 3890 (3427 in Bangladesh and 463 in India) newborns for the analysis involving the exposure of maternal fever in combination with intrapartum asphyxia and 3350 newborns for the analysis of the exposure of PROM and birth asphyxia (Figure 2). The early neonatal death rates among asphyxiated newborn infants without exposure to maternal infection were 160 per 1000 live births in Bangladesh, 408 in India, and 215 in both countries. Asphyxiated infants with signs of maternal fever or PROM had early neonatal death rates of 282 (440 in India and 218 in Bangladesh), and 192 per 1000 live births respectively. Maternal fever was present in 4.5% of the asphyxiated infants (2.7% in Bangladesh, 10.8% in India) while PROM was present in 25.5% of asphyxiated infants in Bangladesh.

Of the 81,778 infants in the general study population, 0.58% (n = 476) of the data were missing for signs of intrapartum asphyxia, 0.01% (n = 12) for maternal fever. In Bangladesh, 1.85% (n = 1181) of the 63,985 newborns present before study exclusions were missing data on PROM. Figure 2 shows the flow of infants from the initial study population to the numbers available for this analysis. Newborns with missing data on intrapartum asphyxia were significantly more likely to have PROM or maternal fever (p < 0.001 for all associations). Infants with missing data on all signs of maternal infection were more likely to experience an early neonatal death (p < 0.001). However because there were so few missing data and bias is unlikely, we do not present differences between the complete and missing data groups.

### Differences between exposed and unexposed newborns

Details of the differences between infants exposed to both asphyxia and PROM or maternal fever and those exposed to asphyxia alone are shown in Table 2 and Table 3 respectively. Asphyxiated infants exposed to both maternal fever or PROM were more likely to have had an early neonatal death (p = 0.004 and p = 0.020 respectively).

**Table 2:** Characteristics of infants with asphyxia and infants with both asphyxia and prolonged rupture of membranes (PROM) in Bangladesh

**Factors Associated with maternal infection**	**Bangladesh**			
	**Overall (n** = **3350)**	**Asphyxia alone (n** = **2502)**	**Asphyxia and PROM (n** = **848)**	**p value**^**a**^
**Newborn health**				
Early neonatal death, *n* (%)				
No	2792 (83.3)	2107 (84.2)	685 (80.8)	0.020
Yes	558 (16.7)	395 (15.8)	163 (19.2)	
**Signs of maternal infection**				
Fever up to three days prior to delivery, *n* (%)				
No	3230 (96.4)	2420 (96.7)	810 (95.5)	0.103
Yes	120 (3.6)	82 (3.3)	38 (4.5)	
Missing				
Vaginal smell up to three days prior to delivery, *n (%)*				
No	2570 (76.7)	1945 (77.7)	625 (73.7)	<0.001
Yes	109 (3.3)	52 (2.1)	57 (6.7)	
Missing	671 (20.0)	505 (20.2)	166 (19.6)	
**Maternal characteristics**				
Maternal education, *n* (%)				
No education	711 (21.2)	525 (21.0)	186 (21.9)	0.436
Primary	1179 (35.2)	896 (35.8)	283 (33.4)	
Secondary	1460 (43.6)	1081 (43.2)	379 (44.7)	
Maternal reading ability, *n* (%)				
Unable to read	862 (25.7)	632 (25.3)	230 (27.1)	0.038
Reads with difficulty	667 (19.9)	525 (21.0)	142 (16.8)	
Reads with ease	1817 (54.2)	1343 (53.7)	474 (55.9)	
Missing	4 (0.1)	2 (0.1)	2 (0.2)	
Maternal age in years, *n* (%)				
<20	797 (23.8)	625 (25.0)	172 (20.3)	0.074
20–29	2014 (60.1)	1482 (59.2)	532 (62.7)	
30–39	506 (15.1)	369 (14.8)	137 (16.2)	
40+	32 (1.0)	25(1.0)	7 (0.8)	
Missing	1 (0.0)	1 (0.0)	0 (0.0)	
Household assets, *n* (%)				
All	1288 (38.5)	954 (38.1)	334 (39.4)	0.552
Some	917 (27.4)	697 (27.9)	220 (25.9)	
None	1145 (34.2)	851 (34.0)	294 (34.7)	
Parity, *n* (%)				
1	1623 (48.5)	1194 (47.7)	429 (50.6)	0.580
2	724 (21.6)	551 (22.0)	173 (20.4)	
3	445 (13.3)	331 (13.2)	114 (13.4)	
4	251 (7.5)	195 (7.8)	56 (6.6)	
5	154 (4.6)	119 (4.8)	35 (4.1)	0.580
6	153 (4.6)	112 (4.5)	41 (4.8)	
**Antenatal period**				
Number of antenatal care visits, *n* (%)				
0	882 (26.3)	688 (27.5)	194 (22.9)	0.067
1	674 (20.1)	507 (20.3)	167 (19.7)	
2	614 (18.3)	453 (18.1)	161 (19.0)	
3	486 (14.5)	360 (14.4)	126 (14.9)	
4	689 (20.6)	490 (19.6)	199 (23.5)	
Missing	5 (0.2)	4 (0.2)	1 (0.l)	
Bleeding during pregnancy, *n* (%)				
No	3182 (95.0)	2401 (96.0)	781 (92.1)	<0.001
Yes	166 (5.0)	100 (4.0)	66 (7.8)	
Missing	2 (0.1)	1 (0.0)	1 (0.1)	
**Delivery period**				
Preterm birth, *n* (%)				
Baby born at term	3004 (89.7)	2250 (89.9)	754 (88.9)	0.565
Baby born after less than 9 months gestation	341 (10.2)	249 (10.0)	92 (10.9)	
Missing	5 (0.2)	3 (0.1)	2 (0.2)	
Baby delivered by skilled delivery attendant, *n* (%)^b^				
Yes	2338 (69.8)	1814 (72.5)	524 (61.8)	<0.001
No	1002 (29.9)	681 (27.2)	321 (37.9)	
Missing	10 (0.3)	7 (0.3)	3 (0.4)	
Institutional delivery, n (%)				
Yes	2458 (73.4)	1901 (76.0)	557 (65.7)	<0.001
No	889 (26.5)	598 (23.9)	291 (34.3)	
Missing	3 (0.1)	3 (0.1)	0 (0.0)	
Excessive bleeding during delivery, *n* (%)				
No	3234 (96.5)	2425 (96.9)	809 (95.4)	0.036
Yes	116 (3.5)	77 (3.1)	39 (4.6)	
Malpresentation at birth				
No	2858 (85.3)	2162 (86.4)	696 (82.1)	0.001
Yes	279 (8.3)	202 (8.1)	77 (9.1)	
Missing	213 (6.4)	138 (5.5)	75 (8.8)	
Type of delivery				
Normal, vaginal	3043 (90.8)	2302 (92.0)	741 (87.4)	<0.001
Vaginal, assisted	83 (2.5)	59 (2.4)	24 (2.8)	
Caesarean	224 (6.7)	141 (5.6)	83 (9.8)	

^a^p value obtain through the use of chi-squared test or Fisher’s exact test.

^b^Doctor, nurse, or trained midwife.

**Table 3:** Comparison between newborns with asphyxia and newborns with asphyxia and maternal fever in Bangladesh and India

**Factors Associated with maternal infection**	**Pooled dataset (n = 3890)**			**Bangladesh (n = 3427)**			**India (n = 463)**		
	**Hypoxia alone (n = 3716)**	**Fever and hypoxia (n = 174)**	**p value**^**a**^	**Hypoxia alone (n = 3303)**	**Fever and hypoxia (n = 124)**	**p value**^**a**^	**Hypoxia alone (n = 413)**	**Fever and hypoxia (n = 50)**	**p value**^**a**^
**Newborn health**									
Early neonatal death, *n* (%)									
No	2997 (80.7)	125 (71.8)	0.004	2752 (83.3)	97 (78.2)	0.137	245 (59.3)	28 (56.0)	0.652
Yes	719 (19.4)	49 (28.2)		551 (16.7)	27 (21.8)		168 (40.7)	22 (44.0)	
**Signs of maternal infection**									
Prom, *n* (%)									
No	na^b^	na	na	2420 (73.3)	82 (66.1)	0.203	na	na	na
Yes	na	na		810 (24.5)	38 (30.7)		na	na	
Missing				73 (2.2)	4 (3.2)				
**Maternal characteristics**									
Maternal education, *n* (%)									
No education	973 (26.2)	69 (39.7)	<0.001	689 (20.9)	33 (26.6)	0.003	284 (68.8)	36 (72.0)	0.735
Primary	1177 (31.7)	59 (33.9)		1150 (34.8)	55 (44.4)		27 (6.5)	4 (8.0)	
Secondary	1566 (42.1)	46 (26.4)		1464 (44.3)	36 (29.0)		102 (24.7)	10 (20.0)	
Maternal reading ability, *n* (%)									
Unable to read	1129 (30.4)	77 (44.3)	<0.001	839 (25.4)	39 (31.5)	0.092	290 (70.2)	38 (76.0)	0.688
Reads with difficulty	675 (18.2)	34 (19.5)		647 (19.6)	31 (25.0)		28 (6.8)	3 (6.0)	
Reads with ease	1908 (51.4)	63 (36.2)		1813 (54.9)	54 (43.6)		95 (23.0)	9 (18.0)	
Missing	4 (0.1)	0 (0.0)		4 (0.1)	0 (0.0)		0 (0.0)	0 (0.0)	
Maternal age in years, *n* (%)									
<20	859 (23.1)	45 (25.9)	0.086	782 (23.7)	33 (26.6)	0.039	77 (18.6)	12 (24.0)	0.449
20–29	2242 (60.3)	90 (51.7)		2004 (60.7)	60 (48.4)		238 (57.6)	30 (60.0)	
30–39	555 (14.9)	36 (20.7)		485 (14.7)	29 (23.4)		70 (17.0)	7 (14.0)	
40+	37 (1.0)	3 (1.7)		31 (0.9)	2 (1.6)		6 (1.5)	1 (2.0)	
Missing	23 (0.6)	0 (0.0)		1 (0.0)	0 (0.0)		22 (5.3)	0 (0.0)	
Household assets, *n* (%)									
All	1367 (36.8)	43 (24.7)	<0.001	1289 (39.0)	34 (27.4)	<0.001	78 (18.9)	9 (18.0)	0.351
Some	1173 (31.6)	51 (29.3)		918 (27.8)	24 (19.4)		255 (61.7)	27 (54.0)	
None	1176 (31.7)	80 (46.0)		1096 (33.2)	66 (53.2)		80 (19.4)	14 (28.0)	
Parity, *n* (%)									
1	1807 (48.6)	68 (39.1)	<0.001	1618 (49.0)	49 (39.5)	<0.001	189 (45.8)	19 (38.0)	0.324
2	798 (21.5)	34 (19.5)		717 (21.7)	24 (19.4)		81 (19.6)	10 (20.0)	
3	476 (12.8)	30 (17.2)		434 (13.1)	20 (16.1)		42 (10.2)	10 (20.0)	
4	275 (7.4)	11 (6.3)		246 (7.5)	7 (5.7)		29 (7.0)	4 (8.0)	
5	173 (4.7)	9 (5.2)		148 (4.5)	8 (6.5)		25 (6.1)	1 (2.0)	
6	187 (5.0)	22 (12.6)		140 (4.2)	16 (12.9)		47 (11.4)	6 (12.0)	
**Antenatal period**									
Number of antenatal care visits, *n* (%)									
0	1007 (27.1)	48 (27.6)	0.672	868 (26.3)	34 (27.4)	0.922	139 (33.7)	14 (28.0)	0.227
1	719 (19.4)	42 (24.1)		658 (19.9)	28 (22.6)		61 (14.8)	14 (28.0)	
2	672 (18.1)	27 (15.5)		605 (18.3)	19 (15.3)		67 (16.2)	8 (16.0)	
3	543 (14.6)	23 (13.2)		481 (14.6)	19 (15.3)		62 (15.0)	4 (8.0)	
4	769 (20.7)	34 (19.5)		686 (20.8)	24 (19.4)		83 (20.1)	10 (20.0)	
Missing	6 (0.2)	0 (0.0)		5 (0.2)	0 (0.0)		1 (0.2)	0 (0.0)	
Bleeding during pregnancy, *n* (%)									
No	3545 (95.4)	165 (94.8)	0.886	3142 (95.1)	115 (92.7)	0.449	403 (97.6)	50 (100.0)	0.266
Yes	169 (4.6)	9 (5.2)		159 (4.8)	9 (7.3)		10 (2.4)	0 (0.0)	
Missing	2 (0.1)	0 (0.0)		2 (0.1)	0 (0.0)		0 (0.0)	0 (0.0)	
**Delivery period**									
Preterm birth, *n* (%)									
Baby born at term	3271 (88.0)	145 (83.3)	0.174	2955 (89.5)	110 (88.7)	0.846	316 (76.6)	35 (70.0)	0.595
Baby born less than 9 months gestation	433 (11.7)	28 (16.1)		342 (10.4)	14 (11.3)		91 (22.0)	14 (28.0)	
Missing	12 (0.3)	1 (0.6)		6 (0.2)	0 (0.0)		6 (1.5)	1 (2.0)	
Baby delivered by skilled attendant, *n* (%)^c^									
No	2574 (69.3)	113 (64.9)	0.346	2282 (69.1)	85 (68.6)	0.799	292 (70.7)	28 (56.0)	0.034
Yes	1131 (30.4)	61 (35.1)		1010 (30.6)	39 (31.5)		121 (29.3)	22 (44.0)	
Missing	11 (0.3)	0 (0.0)		11 (0.3)	0 (0.0)		0 (0.0)	0 (0.0)	
Institutional delivery, n (%)									
No	2708 (72.9)	119 (68.4)	0.392	2399 (72.6)	89 (71.8)	0.921	309 (74.8)	30 (60.0)	0.025
Yes	1005 (27.1)	55 (31.6)		901 (27.3)	35 (28.2)		104 (25.2)	20 (40.0)	
Missing	3 (0.1)	0 (0.0)		3 (0.1)	0 (0.0)		0 (0.0)	0 (0.0)	
Excessive bleeding during delivery, *n* (%)									
No	3578 (96.3)	151 (86.8)	<0.001	3187 (96.5)	116 (93.6)	0.085	391 (94.7)	35 (70.0)	<0.001
Yes	138 (3.7)	23 (13.2)		116 (3.5)	8 (6.5)		22 (5.3)	15 (30.0)	
Malpresentation at birth, n (%)									
No	3171 (85.3)	144 (82.8)	0.336	2798 (84.7)	104 (83.9)	0.326	373 (90.3)	40 (80.0)	0.085
Yes	291 (7.8)	19 (10.9)		271 (8.2)	14 (11.3)		20 (4.8)	5 (10.0)	
Missing	254 (6.8)	11 (6.3)		234 (7.1)	6 (4.8)		20 (4.8)	5 (10.0)	
Type of delivery, n (%)									
Normal, vaginal	3378 (90.9)	159 (91.4)	0.858	2979 (90.2)	114 (91.9)	0.474	399 (96.6)	45 (90.0)	0.067
Vaginal, assisted	88 (2.4)	3 (1.7)		84 (2.5)	1 (0.8)		4 (1.0)	2 (4.0)	
Caesarean	250 (6.7)	12 (6.9)		240 (7.3)	9 (7.3)		10 (2.4)	3 (6.0)	

^a^p value obtain through the use of a chi-squared test or Fisher’s exact test.

^b^Not applicable: data were not collected in the study.

^c^Doctor, nurse, or trained midwife.

na, not available.

### The double hit hypothesis: maternal infection, intrapartum asphyxia and risk of early neonatal death

Table 4 shows unadjusted and adjusted odds ratios for the associations using the pooled data from Bangladesh and India, as well as separately for each site. Unadjusted analyses using the pooled data show evidence to support the double hit hypothesis: there was a significant increased likelihood of early neonatal death in births with maternal fever and intrapartum asphyxia compared to those with signs of intrapartum asphyxia alone (OR: 1.63, 95% CI: 1.12 – 2.65). Taking PROM as the sign of maternal infection, (only data available from Bangladesh), the association was also significant (OR 1.24, 95% CI: 1.01 – 1.53). Adjustment for possible confounders had little effect on the estimates for the association between maternal fever and asphyxia with early neonatal mortality (AOR: 1.54, 95% CI: 1.04 – 2.27). The association for maternal fever were broadly similar in Bangladesh and India. The adjusted odds ratio with PROM changed to AOR: 1.28, 95% CI 1.03 – 1.59.

**Table 4:** **Unadjusted and adjusted odds ratios (95**% **CI) comparing early neonatal death among infants with birth asphyxia and maternal infection and infants with intrapartum asphyxia alone**

**Maternal sign of infection**	**Bangladesh and India**^**d**^	**Bangladesh**	**India**
	**(odds ratio, 95% CI)**	**(odds ratio, 95% CI)**	**(odds ratio, 95% CI)**
Unadjusted analysis			
Fever	1.63 (1.12 – 2.65)	1.62 (1.03 – 2.54)	1.73 (0.64 – 3.48)
PROM	^a^	1.24 (1.01 - 1.53)	^a^
Adjusted analysis			
Fever^c^	1.54 (1.04 - 2.27)	1.48 (0.93 - 2.36)	1.90 (0.88 – 4.15)
PROM^c^	^a^	1.28 (1.03 - 1.59)	^a^
Unadjusted cause of death analysis			
Fever	1.89 (1.02– 3.49)	1.82 (0.67 – 4.92)	2.08 (0.94 – 4.65)
Prom	^a^	1.42 (1.10 – 1.83)	^a^
Adjusted cause of death analysis			
Fever^c^	1.65 (0.84 – 3.23)	^b^	^b^
PROM^c^	^a^	1.52 (1.15 – 2.02)	^a^

^a^Data not collected on PROM in India.

^b^Models would not converge.

^c^Adjusted for maternal education, parity, maternal age, number of antenatal visits, malpresentation at delivery, type of delivery, household assets, delivery by a skilled birth attendant, delivery at an institution, and intervention or control allocation and clustering.

^d^Combined datasets also adjusted for study site into account.

### Findings from cause of death data

Results using InterVA-derived cause of death data are summarised in Table 4. Using the outcome of an early neonatal death due to birth asphyxia and adjusting for clustering and previously identified confounders, the adjusted odds ratio supported the double hit hypothesis in relation to PROM: there was a significantly increased odds of an early neonatal death due to birth asphyxia in newborns with exposure to PROM (AOR: 1.52, 95% CI 1.15 – 2.02). Using similar methods, the unadjusted odds ratio supported the double hit hypothesis in relation to exposure of fever, however this did not hold true for the adjusted analysis: there was no significant increased odds of an early neonatal death due to birth asphyxia in newborns with exposure to maternal fever (AOR: 1.65, 95% CI: 0.84 – 3.24). According to the verbal autopsy data for our study cohort, intrapartum asphyxia accounted for 59% of early neonatal deaths, neonatal pneumonia accounted for 14%, and prematurity 10%.

## Discussion

We have shown that newborn infants in rural Bangladeshi and Indian who were exposed to signs of maternal infection as indicated by PROM as well as intrapartum asphyxia were at increased risk of early neonatal mortality compared to those who only had signs of asphyxia. Other clinical studies have suggested a similar association, but ours is the first large scale population study to test the hypothesis [17-22]. A cohort study from a large hospital population in Kathmandu Nepal, demonstrated that antepartum maternal fever was a significant predictor of intrapartum-related neonatal death after adjusting for other factors [16].

Collecting information on complications that occur in the antepartum and delivery periods in rural, low resource settings is wrought with difficulties due to reporting bias, as most deliveries occur in the home without a skilled birth attendant. However, until facility-based deliveries become the norm, surveillance data collected using similar methodology, as in this study, will need to rely on self-reporting by interviews after delivery [32]. Our data relied on a mother’s or close relative’s recall of events in delivery and the antepartum period. There have been attempts to validate such questionnaires in the past, with one study indicating that medically diagnosed conditions occurring during delivery, such as neonatal condition at five minutes, PROM, and maternal fever, are often over-estimated when using questionnaires relying on a women’s recall, especially when this event is rare [33]. In this instance, overestimation of the presence of a complication would lead to underestimation or a diluted estimate for the effect of maternal infection in asphyxiated infants. Recall bias following a neonatal death or significant morbidity could lead to either under- or over-reporting of maternal signs of infection and symptoms of intrapartum asphyxia, and therefore to over or under-estimation of the effect sizes. For instance, if a neonatal death occurs the mother may be searching for explanations as to why this happened, and this may lead to systematic differences in the way these women answered questions compared to women whose newborns survived.

Misclassification bias can occur inadvertently for the indication of maternal infection, as factors such as an incompetent cervix or abnormal presentation of the foetus can induce PROM. Furthermore, after membrane rupture, serious consequences such as prolapsed umbilical cord may increase the risk of intrapartum asphyxia [34]. Similarly, ‘fever’ may indicate a wide range of infectious diseases including influenza, pneumonia, malaria, and typhoid fever, not all of which always affect the fetus [28]. Data was also available on “vaginal smell three days prior to delivery”. In this instance, it was thought the potential for misclassification bias was too great, so this was not assessed further. If maternal signs of infection have been over-estimated due to misclassification bias, assuming the double hit hypothesis holds true, this would under-estimate a positive association of maternal infection for early neonatal death in asphyxiated infants. Additionally, a diagnosis relying on the presence or absence of a symptom has been shown to have a higher sensitivity than a diagnosis that relies on a question indicating the severe end of a normal continuum, such as excessive bleeding [32]. For this reason, we feel that PROM is less subject to recall bias than maternal fever.

Differences were noted in the prevalence of signs of maternal infection and intrapartum asphyxia between Bangladesh and India. In Bangladesh, approximately 5% of newborn infants exhibited signs of intrapartum asphyxia, compared with 2.5% in India. In Bangladesh, higher use of birth attendants trained specifically to recognize signs of birth asphyxia may have made mothers more aware of signs of the event that account for this difference. The combination of maternal fever and intrapartum asphyxia led to higher mortality risk in Bangladesh compared to India, perhaps due to under-reporting of intrapartum asphyxia in India. If this were the case, then our pooled analysis underestimates the true effect of the double hit hypothesis. In India, approximately 4.8% of all newborns were exposed to maternal fever for up to three days prior to delivery compared to 1.7% in Bangladesh, possibly due to the higher incidence of malaria [35]. The percentage of women with PROM (16.6%) observed in Bangladesh was slightly higher than in similar populations elsewhere. Ellis *et al.* in Nepal found 11% PROM rates in deliveries where infants exhibited signs of intrapartum asphyxia [17]. In a prospective community study assessing risk factors for birth asphyxia in Nepal, 14% of asphyxiated infants had mothers with prolonged rupture of membranes longer than twenty-four hours [11]. There is great variation in the prevalence of reported signs of both intrapartum asphyxia and maternal fever between Bangladesh and India. This variation is potentially due to differences in the way these questions were administered in each country, or could be due to genuine differences in morbidity patterns.

Given the limitations of this analysis due to inherent biases associated with observational data, we used verbal autopsy reports to identify newborns that were likely to have died due to intrapartum asphyxia. Use of the InterVA method ensured that causes of death were standardized between study settings and over time (a significant advantage in the current study) and classification of asphyxia cases based on probabilistic reasoning relating to multiple causes may overcome misclassification bias associated with lay-reports and alternative methods of verbal autopsy interpretation. The verbal autopsy data validated our findings to some extent, in that the risk of an early neonatal death due to intrapartum asphyxia was significantly greater when the neonate was exposed to PROM compared to an unexposed newborn. However, when using maternal fever as the surrogate for maternal infection, there was no significant increased risk of death due to an intrapartum event. The lack of an effect in the exposure of maternal fever could have occurred due to a low sensitivity of this marker of maternal infection and the potential for misclassification bias previously mentioned. However, the fact that the association between PROM and early neonatal death due to intrapartum asphyxia was greater than the association between PROM and an early neonatal death alone, adds weight to the double hit hypothesis.

Our population-based results support, but do not prove, the double-hit hypothesis that an insult such as maternal infection during critical periods of neural development sets up a predisposition toward severe adverse outcomes when exposed to asphyxia [19]. It might be argued that the two events of infection and asphyxia act independently but additively to affect early newborn death rather than through a double-hit interaction. Further studies are needed to understand the biological mechanisms behind the double hit hypothesis in similar low-resource settings, and follow-up to assess the risk of disabilities from a double hit compared with asphyxia alone.

Our findings beg the important policy question whether antibiotic treatment for women with maternal signs of infection should be routinely offered to reduce early neonatal deaths and, possibly, later disability. The use of antibiotics is already advocated for women experiencing preterm premature rupture of membranes [36]. It is also arguable that based on currently available evidence, women who experience prolonged rupture of membranes should be given antibiotics in low-income settings. In addition to treatment women with signs of infection due to bacteria, a recent review of malaria in pregnancy in the Asian context stresses the importance of early detection and treatment of any malaria in pregnancy to prevent the effects of symptomatic disease including stillbirth, intrauterine death, and low birth weight, which as corroborated by a recent hospital study in [37,38]. There is a surprising lack of trial evidence addressing this question in high-income settings. However benefits are likely to be greater for home births in low-income populations, where the risk of newborn infection is far higher [39]. Trials of antibiotics given to mothers with signs of infection, either in the community or upon arrival at hospital, could assess not only early mortality impact, but also the effect on incidence of neurodevelopmental sequelae.

## Conclusion

Our findings support the double hit hypothesis: newborn infants born in rural south Asia showing evidence of intrapartum compromise are more likely to suffer an early neonatal death if the infant’s mother also has evidence of infection. Our finding supports the prompt treatment of mothers exhibiting signs of infection with antibiotics, and raises the question of antibiotic treatment for pregnant women suffering pre-labour rupture of membranes. Benefits are likely to be greater for low-income home births.

## Abbreviations

cRCTs: Cluster randomized controlled trials; PROM: Prolonged rupture of membranes.

## Competing interests

All authors have completed the Unified Competing Interest form at http://www.icmje.org/coi_disclosure.pdf and declare: no support from any organization for the submitted work [or describe if any]; no financial relationships with any organizations that might have an interest in the submitted work in the previous three years [or describe if any], no other relationships or activities that could appear to have influenced the submitted work [or describe if any].

## Authors’ contributions

AC and AI conceptualized the study. AI carried out the initial literature searches and wrote the background and discussion for the first draft of the paper. NS wrote the methods and results, carried out literature searches, was responsible for analysis and subsequent collation of inputs and redrafting. AP edited all drafts of the paper, contributed to the analysis plan and interpretation of results. ME edited all drafts of the paper and contributed to the analysis plan, interpretation of results with particular attention to the background section and conclusions. A.Copas was involved in the analysis plan, editing drafts of the paper, in particular the statistical methods, results and interpretation of the results. EF conducted the verbal autopsy analysis and contributed to the interpretation of findings. KA led the field research in Bangladesh and edited drafts of the paper. PT led the field research in India and edited drafts of the paper. All authors had full access to all the data (including statistical reports and tables) in the study and can take responsibility for the integrity of the data and the accuracy of the data analysis. All authors read and approved the final manuscript.

## Pre-publication history

The pre-publication history for this paper can be accessed here:

http://www.biomedcentral.com/1471-2393/13/245/prepub
